# Supplementary material for: Effects of progessive vs. constant protocol whole-body vibration on muscle activation, pain, disability and functional performance in non-specific chronic low back pain patients: a randomized clinical trial
Source: PeerJ. 2024 Oct 24;12:e18390. doi: 10.7717/peerj.18390 (PMC11512803; doi:10.7717/peerj.18390)
Supplement: Supplemental Information 2 [file peerj-12-18390-s002.docx]

**Key to master chart**

**Group 1**: Constant/fixed protocol Whole-body vibration

**Group 2**: Progressive protocol Whole-body vibration

**Gender 1**: Male

**Gender 2**: Female

**BMI:** Body mass index

**VAS:** Visual Analog scale

**RMDQ**: Rolland Moris disability questionnaire

**PILE:** Progressive isoinertial lifting evaluation

**RA**: Rectus Abdominis

**EO**: External Oblique

**ES**: Erector Spinae

**MF**: Multifidus

**AvgRMS**: average of three repetition of Root mean square obtained in normal activity

**AvgMVIC**: average of three repetition of Root mean square obtained in Maximum voluntary isometric contraction

**%MVIC**: percentage MVIC

**1**: Pre-treatment

**2**: Post-treatment
